# Using social media to support small group learning

**DOI:** 10.1186/s12909-017-1060-7

**Published:** 2017-11-10

**Authors:** Duncan Cole, Emma Rengasamy, Shafqat Batchelor, Charles Pope, Stephen Riley, Anne Marie Cunningham

**Affiliations:** 1 Centre for Medical Education, Cardiff University School of Medicine, 5th Floor, Cochrane Building, University Hospital of Wales, Heath Park, Cardiff, Cardiff, CF14 4XW UK; 2 NHS Wales Informatics Service, Cardiff, UK

**Keywords:** eLearning, Social media, Problem-based learning, Curation, Wiki

## Abstract

**Background:**

Medical curricula are increasingly using small group learning and less didactic lecture-based teaching. This creates new challenges and opportunities in how students are best supported with information technology. We explored how university-supported and external social media could support collaborative small group working on our new undergraduate medical curriculum.

**Methods:**

We made available a curation platform (Scoop.it) and a wiki within our virtual learning environment as part of year 1 Case-Based Learning, and did not discourage the use of other tools such as Facebook. We undertook student surveys to capture perceptions of the tools and information on how they were used, and employed software user metrics to explore the extent to which they were used during the year.

**Results:**

Student groups developed a preferred way of working early in the course. Most groups used Facebook to facilitate communication within the group, and to host documents and notes. There were more barriers to using the wiki and curation platform, although some groups did make extensive use of them. Staff engagement was variable, with some tutors reviewing the content posted on the wiki and curation platform in face-to-face sessions, but not outside these times. A small number of staff posted resources and reviewed student posts on the curation platform.

**Conclusions:**

Optimum use of these tools depends on sufficient training of both staff and students, and an opportunity to practice using them, with ongoing support. The platforms can all support collaborative learning, and may help develop digital literacy, critical appraisal skills, and awareness of wider health issues in society.

## Background

Cardiff University School of Medicine has implemented a new curriculum termed “C21” which reflects the general shift in Undergraduate medical education from the use of didactic lectures towards student-centred small groups. We use a case-based learning (CBL) approach, a modification of problem-based learning [1, 2] where clinical cases are used as the means (or “anchor”) to explore the required learning in context. This approach is used in, and structures, the first 2 years of the course, and continues in a modified form later in the course. Whilst having similarities with problem-based learning (PBL), our approach is more guided.

At the end of the first term, the 300 year 1 students are divided into groups of ten and CBL is introduced using a practice case to explore the educational framework used to run the group sessions. Using the Seven Steps approach [1], the students are introduced to the case scenario in the first session which ultimately generates a series of student-derived learning goals to explore in self-directed learning time during the week. The second session allows the students to share their learning before a case extension introduces the subsequent scenario with further learning outcomes. The case is then “wrapped up” during the final session. The students are supported during the case using plenary lectures, practical classes, community patient contact and clinical skills. Timetabled self-directed learning is essential in this model to allow students to access and read resources relevant to the case, and which address the learning outcomes they have identified during the tutorials.

Student learning is supported by information technology (IT) within our university, primarily in the form of our Virtual Learning Environment (VLE), Learning Central, which is powered by Blackboard. Faculty are aware that students increasingly use the Internet to support their studies and, as reported in the literature, use Google and Wikipedia frequently [3]. The use of the internet can promote student learning, with effects comparable to other instructional methods, e.g. face-to-face, but with the advantage that multi-media materials can be accessed in a time and place convenient to the student [4]. However, there remained a concern among faculty that students may use inaccurate or poor quality online resources, and too readily use sources such as Wikipedia rather than peer-reviewed resources, a concern also reflected in the literature [5, 6].

The use of social media in higher education is also becoming increasingly widespread as online tools that promote collaborative working and discussion become available. Medical students use a variety of social networking sites, and an increasing proportion use such tools for learning and professional purposes [7]. Platforms such as wikis, Facebook, Pinterest and Google + have been used with positive outcomes in diverse learning and teaching applications, for example developing and curating resources on nursing informatics [8]; as an interactive online final year project notebook [9]; and developing medical student professionalism [10]. We therefore considered that embedding social media tools into our course could provide a constructive way to engage our students and promote collaborative activities online as well as in face-to-face teaching.

Faculty considered a range of problems in the context of our new course to which the use of internet-based learning resources and social media were relevant:

1. How do we best support sharing of online resources between students, and between students and staff? Sharing of large lists of web-links by staff provides little information to allow students to select the resource that would be of most value to them.
2. How do faculty ensure students are using the best quality online resources for their learning? We wished to ensure students were directed to reliable, user-friendly resources.
3. How do we encourage students to use social media in a responsible and professional manner? Social media has often been seen in a negative light, particularly when students post inappropriate comments or photographs, and we wished to change this perception and encourage more positive use.

From a student perspective, additional issues were identified. Problems with flexibility and ease of use of our VLE have previously been raised, suggesting that students may prefer to use other platforms. Direction from faculty on which of the many resources available on the internet to focus on i.e. the “information overload” problem [11] had also been raised.

These problems are not confined to our course, but reflect a wider trend in the use of the Internet by staff and students. We recognized that they had become more prominent with the change in curriculum, as the emphasis on student responsibility for their own learning and seeking the information required to understand the case they were studying meant that students had a requirement to search for information. The ubiquity of the internet and ease of use of search tools such as Google means that many turn to the internet first when trying to answer a specific question [3]. This contrasts with “traditional” medical courses, where information is presented more didactically and therefore systematically by faculty, a structure that is also adopted by textbooks, and which arguably requires less searching for information by students.

Our aim, therefore, was to identify appropriate social media tools that would meet student needs on the new C21 course, and to implement and evaluate these in year 1 of the course.

## Methods

### Identification of potential solutions

Following our consideration of the problems stated above, we identified three specific needs for students:

1. To be able to create a record of their learning.
2. To be able to share resources within their CBL groups.
3. To receive feedback from staff on the quality of resources they used.

We identified possible solutions by reviewing supported applications within our VLE and searching the internet for platforms that might be suitable for our needs, and reviewing these in detail. We were already aware that several members of staff used web-based applications to support their teaching, and we also reviewed the suitability of these.

Our investigations identified three main classes of software that could meet one or more of the needs identified. These were wikis, online multi-page documents that can be updated by all group members; curation platforms, which allow online content relevant to a particular audience to be gathered in the same place, with the intent to link the items in some way and to provide commentary and/or a narrative that gives additional insight from the curator; and social media discussion platforms, such as Facebook (see Table 1 for more details). We recognised that Facebook would likely be used by students regardless of our suggestions, and made an active decision not to mandate the use of a specific platform, but to provide University-supported options in addition to those that students might usually select. Blogs were not considered a viable option as they did not provide facilities for easy collaboration.

**Table 1:** Analysis of social media options

Type of platform	Platform(s) assessed	Strengths	Weaknesses
Virtual Learning Environment (VLE)	Blackboard	University supported; used by all students; other course material is readily available; links to web-based resources can be added; discussion forums available	Limited ability for students to add web links; experience shows discussion forums usually rarely used by students; interface is not as user friendly as web-based applications; sharing of resources between students is difficult
Social media discussion	Facebook	Well known and used by students; able to post links directly into a feed and comment on it; can see who has viewed the post; groups can be set up with a variety of privacy options	Students use Facebook for their personal social life – may not wish to use for work. Resources not collected in one easy-to-access place. Group collaboration on same document is not intuitive and auditable. Not supported by university and cannot integrate with VLE.
Wiki	Campus Pack Wiki	Available as an add-on in the VLE. Good for group collaboration tasks on same document; edits are auditable – can see who has contributed; can add additional pages easily; allows upload of documents as well as hyperlinks to web-pages	Need to be familiar with the document structure to navigate effectively; sharing of documents does not occur outside of wiki group; accessing can be difficult with multiple steps
Online content curation	Scoop.it Pinterest Del.i.cious	Aesthetically pleasing; easy to add web-based resources to pages via a “bookmarklet” and to add a commentary; can share resources to other social media sites; groups can contribute to same topic pages	Not supported by the university; new tools, so students and staff will not be familiar with how to use them; some integration possible with VLE, but often limited with free and education versions.
Blogs	Cardiff blogs	University supported; can post articles and commentaries with web-links; can comment on posts	Not as “spontaneous” and easy to use for capturing learning as other platforms; too structured and needs time to learn how to use; usually public-facing

Our review and assessment of the various options highlighted that different tools would be helpful for specific problems, and that curation platforms, wikis and social media communication tools such as Facebook were complementary. Specifically, curation platforms allow students and staff to organise online resources effectively and provide a forum for the discussion of quality, whereas wikis were highly effective for allowing students to collaborate on specific items of work, such as individual learning outcomes. These tools did facilitate some discussion, but usually related to the material or resource presented, which left a need for a more generic communication tool. Ideally, these tools would be linked, so that curated resources could be linked to the relevant wiki page, but we could not identify a means through which this could be achieved.

Following our analysis (see Table 1), we decided to offer two solutions - the curation platform Scoop.it, and the Campus Pack wiki. Under the terms of the Scoop.it educational license all activity within the curation platform was publically viewable. At additional cost an enterprise solution would have allowed Scoop.it pages to be private, but we thought that this would not give students the same opportunity to develop a professional digital identity by considering the content that would be attributable to them both individually and as a group. We chose to use the Campus Pack wiki as the range of functions was superior to Blackboard’s own tool.

### Implementation

We implemented our chosen social media solutions, Scoop.it and the Campus Pack wiki, with year 1 medical students. Students were randomly assigned to 30 CBL groups by the year administrators, with 10 students per group. Faculty involved in the implementation were CBL tutors (one per CBL group) and CBL case leads for year 1, as well as the core implementation team which included academic leads (AMC, DC) and the Phase 1 Director (SR).

We implemented Scoop.it and the Campus Pack wiki for the first CBL case, which followed the practice case. At the start of the second semester students were asked how they had communicated during the practice case. The majority had used Facebook but 58% said that they would like the option of using alternative platforms for university work. They were then introduced to the wiki and curation platform. We emphasised that the selection of tools to use, including those we had not specifically introduced, remained the choice of individual groups.

**Fig. 1:**
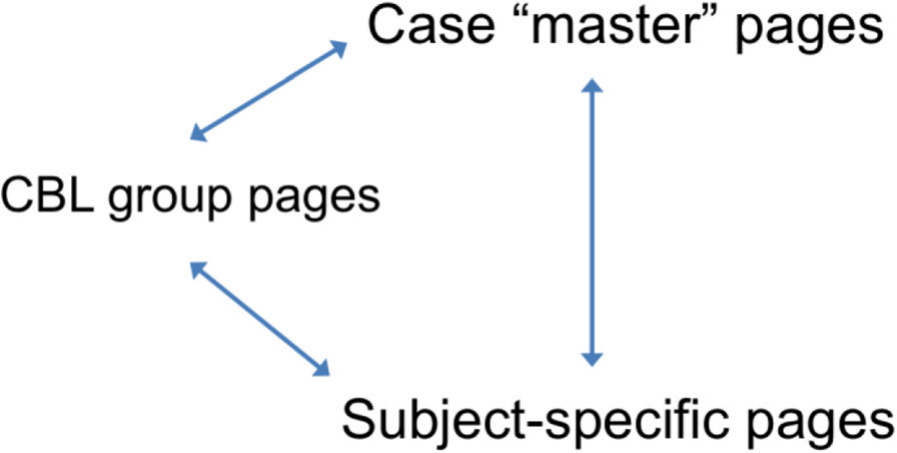
Scoop.it topic page structure. Pages were set up for each CBL case (the Master Page), each student CBL group, and for specific subjects (eg Biochemistry, Reproductive Medicine). The arrows indicate that posts to each page could be “re-scooped” to any of the other pages

Each of the 30 CBL groups had a Scoop.it page set up, and all members of the group were invited to join as co-curators. We set up a case “master” page for each of the 6 cases to run during the year, and several subject pages, e.g. biochemistry, physiology, anatomy, renal medicine (see Fig. 1). Plenary sessions were run to introduce students to Scoop.it, explain how it works, and how to use it in CBL. The publically viewable nature of the platform was emphasised, and students were encouraged to consider this when using it. Staff facilitators also had a training session, and an online forum for discussion with facilitators was started. In addition, we set up a project blog, where further information could be found. We posted screencasts demonstrating how to use Scoop.it and linked to the Scoop.it website FAQs. We invited each student small group to curate their own page and “post” resources. These were reviewed by staff who selected particularly useful content and shared (‘re-scooped’) items of content to whole year topic pages for each two-week case. Students were also encouraged to select content from the whole year case pages and share this on their group pages.

The Campus Pack wiki was set up for each CBL group within our VLE (Blackboard) and students were also introduced to this in the plenary session. Unlike Scoop.it, this was accessible only by the group members and facilitator.

The tools remained available for use for the duration of the first year, and remained active following this, in order that students could refer to their created and curated resources as needed for the duration of the course. Our use of the VLE (Blackboard) continued as we had used it on the previous course, acting as a repository of learning materials, timetables, course handbooks, etc. Staff were not asked to post their learning materials on the new social media platforms, but were provided with the option of posting website links with commentaries to the subject-specific and case master pages if they wished.

### Evaluation

We used several approaches to understand the how these tools were used in practice, including surveys, metrics on posts and views of topic pages on Scoop.it, and group activities on the wiki. The survey was run during the second CBL case; students were informed of the purpose of the evaluation and recruited via email. GoogleDocs was used to run the survey; all responses were anonymous. We asked students which CBL group they were in; which tools they and their group were using to communicate, learn and share; what was good about their chosen approach, and what could be improved; what would help them use the tools better; and any other comments they had. The Chair of the School of Medicine Research Ethics Committee reviewed the study and confirmed that as an evaluation of a component of our programme it did not require formal ethical review.

## Results

The online survey in the spring semester allowed us to understand some of the problems students were encountering with the use of technology to support their learning, and where there had been notable successes. Seventy-one students responded to the survey, representing 28 of the 30 groups; some of the comments are given in Table 2. It was evident from this survey, and from informal feedback from CBL facilitators, that most groups were using Facebook in addition to the tools we had made available. Students noted Facebook to be more easily accessible than Scoop.it and the wiki through apps on tablets and smartphones. Since using Facebook was what most group members ‘were used to doing’ it was the obvious first choice for many for online communication outside the CBL classroom. In addition to familiarity, Facebook also had features that were useful for group work such as being able to quickly post a message and seeing who had seen it (via the “just seen” feature).

**Table 2:** Summary of student comments on use of social media platforms offered

Platform	Pros	Cons
General/all	• Technology provides a quick and easy way to get in touch and share documents over the internet from home which is time-saving and convenient. • Able to update learning outcomes and notes immediately in the case • Sharing documents gives different perspectives • Builds relationships within the group and promotes teamwork	• Everyone putting resources in different places • Tendency to copy and paste and not filter or process information
Facebook	• Lots of communication • Instant/fast • More likely to check for updates • Everyone has the app and is on regularly • Able to easily share links • Can host documents • Can quickly get a message out about something	• Sometimes group moves from university-related work to personal • Hard to organise (no tagging or folders so can’t be structured) • More difficult to review past information beyond a certain point
Scoop.it	• Main case page useful • Posting resources to the group page saves time • Information is put up quickly • Resources are all in one place • Use in sessions to see what has been added; can upload session learning outcomes	• Not everyone in the group understands how to use Scoop.it • Some group members don’t view the group page • Can’t (or don’t know how to) scoop some things, eg Word or pdf documents, textbook pages • Confusing to search
Wiki	• Good reference point for the learning outcomes and session notes • Use in sessions – can see what has been added, scribe can add notes, can project for everyone to see • Everyone can add and edit – can produce really comprehensive revision notes	• No alert system for updates • Can be difficult to use

**Fig. 2:**
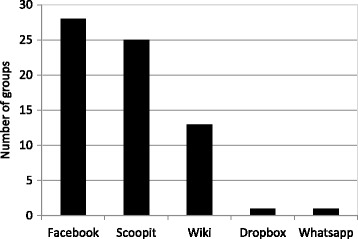
Number of student groups using each social media platform to support CBL

Monitoring of the metrics on the use of the platforms, as well as CBL facilitator feedback and survey data, revealed that different groups adopted different arrangements for collaborative working, and used the various electronic platforms to support this in a way that facilitated their working. The reasons why groups chose to use a particular combination of tools was not explored further. The most widely used platform was Facebook with 28 of 30 groups reporting some usage (Fig. 2). Some elected to use wikis predominantly, rather than Facebook, as it was perceived to be a more professional tool. Others made good use of Scoop.it and liked the fact that all the resources were in one place. The pattern of use within the groups did not change between the interim analysis and the end of year analysis, i.e. those groups that used Scoop.it heavily early on continued to use it through the year, and groups that did not use it early on did not then begin to use it part way through the year. This suggested that the groups decided on a strategy early on and stuck with it for the remainder of the academic year.

We collected interim (after case 3) and end-of-year (after case 6) data on the use of Scoop.it (see Table 3, data from after case 6). Twenty-five of the 30 groups made some use of the platform (Fig. 2), with 91 students (approximately a third of the year) posting at least one resource to their group’s webpage (Table 3). Limitations using Scoop.it were noted in the survey and from informal feedback: in a blended learning environment, not all resources are electronic, and students found it was difficult to refer to textbooks, which was something that could be more easily achieved using the wiki. Students did like the user-friendly layout of Scoop.it, and the ease with which web-pages could be captured. The faculty-curated pages were also highly valued, both for the case and specific subjects.

**Table 3:** Scoop.it usage by students in CBL groups, at the end of case 6

	Scoops	Curators	Views
Total (all groups)	704	91	3144
Min (group)	0	0	14
Max (group)	97	9	380
Mean (per group)	23	3	105

Use of the wiki was less widespread, largely because many found it difficult to use. Some groups adopted it enthusiastically, and found it a useful way to record their agreed learning outcomes and share photos taken of flipcharts in the face-to-face sessions. Some groups used Scoop.it for this as well, although comparatively few. Individuals also found the wiki useful for revision, as group work was available in a directory-like format. In contrast, content in Facebook is essentially chronological with the content most recently engaged with appearing first. Tagging in Scoop.it does allow a more structured approach, but was very rarely used by students.

Although we felt that choice was important for the students, in some cases this appeared to cause confusion, particularly where there was no agreed strategy within the CBL group. This resulted in material intended for the whole group being posted in different places by different members of the group.

Staff engagement with the various platforms was variable, with some engaged and following student activity, and others not engaging at all. Most student groups kept their Facebook groups private, and did not invite their tutors to be members. Web-based resources were reviewed and used by several groups in their face-to-face CBL sessions, and staff engaged well with their presentation in this format.

A review of the content “scooped” by students revealed that in addition to useful learning resources relevant to the case that was being studied, resources that were not directly relevant but might be useful for revision purposes were being scooped and identified as such. Students also identified topical issues in the media that related to the case they were studying.

## Discussion

Our aim with the tools we elected to offer our students were intended to support their learning in CBL, and help them organise their learning and resources. The use demonstrated by the students suggests that some groups, but not all, found Scoop.it and the Campus Pack Wiki to be useful. That the majority of groups did use social media tools (including Facebook) to share resources indicated that our second aim, to provide a means for students to easily share resources, was met. Our first aim, to provide a record of student learning, was met in part: although these tools did provide a record they were not integrated into a single online student learning tool.

From our evaluation, we concluded that the different platforms offered complementary features useful to student learning. Scoop.it was useful for capturing and sharing useful online resources, whereas the wiki had features that were more useful for keeping records of CBL group meetings and organizing a wide variety of information. The implementation phase revealed a need for a tool to facilitate group discussion outside of the classroom, and Facebook was used for this purpose by most groups. We provided choice in the tools students could use, and we noted that groups adopted a way of working that suited them early on. However, this did mean that students electing not to use Scoop.it may have missed out on useful resources.

With the introduction of social media tools, we aimed to help develop in students a professional approach to social media. This is a challenging aim, and the optimal strategy for developing this in medical students is not clear. Other studies have suggested that the perception of a professional space with faculty oversight can help develop this [10] and we aimed to use Scoop.it in this way, but issues such as the use of an open technology may have deterred some students. For example, the literature suggests that those with dyslexia may be unwilling to post a public comment [9]. Faculty may also regard the public nature of many of the available curation tools to be undesirable if students are posting in an unsupervised manner, and may wish to review and approve posts before they are publically viewable, as has been done in other studies [8]. However, a review of student contributions in our study indicated that where students used Scoop.it, they did interact positively and in a professional manner, although more training and guidance and a clear governance structure would be needed for a sustainable implementation, as reviewing posts from a year of 300+ students is a considerable task.

We noted in our evaluation that students and staff did not make full use of the tools provided, and potentially useful features, such as tagging which can help organise resources, were almost never used. This raises the issues of digital literacy and adequate training. Digital literacy is a broad concept, and covers technical skills required to operate a computer, skills required to use and navigate software and the internet, skills required to find, select, and organize information on the internet, and skills required to successfully manage online interactions [12]. We selected tools we felt were relatively easy to use, and did provide some introductory training, but further face-to-face support would likely have been beneficial during the implementation period. As only a small number of staff were fully trained, setting up, managing, and monitoring the use of Scoop.it and the wiki generated a significant workload and resulted in problems with sustainability. A greater emphasis on supporting staff and students, particularly in the initial introductory phase would likely have improved use.

A great benefit of the use of technology such as this is that it promotes collaborative working in an online space, which maintains the pedagogical principles of CBL. The approach we have taken to resource collection and organization, i.e. resources identified and selected by students, and reviewed by both faculty and students, can be thought of as a form of crowd-sourcing, or perhaps more accurately class-sourcing [13]. The collaborative nature of this has the potential to be a powerful way of identifying high-quality resources of relevance to student learning in a specific context. This approach also results in improved faculty awareness of the resources students find useful, and enables faculty to identify what additional resources may need to be developed in-house to complement the open education resources available on the internet. It also provides the opportunity for faculty to critically appraise websites they may not have known were being used by students. Another benefit, which we did not initially anticipate, was students linking their learning and issues raised in the case to current affairs and wider health issues. This helped broaden student horizons by encouraging them to recognize the relevance of their learning to the big issues facing society.

## Conclusion

Social media can be used constructively to complement CBL. Benefits are apparent for both students and faculty, and include provision of a learning record, sharing of useful resources, linking to current affairs, and making explicit the learning resources students are using. However, issues of sustainability, public vs closed group posting, and ensuring adequate digital literacy to maximally benefit from using such tools were raised during our implementation. These may be addressed through a process of hands-on training, mentoring, and careful selection of online tools so they are acceptable to all and provide an integrated and useful means to enhance learning.
